# AGING AND WELL-BEING: TESTING MINDFULNESS AS A MEDIATOR AND RESILIENCE AS A MODERATOR IN A NIGERIAN SAMPLE

**DOI:** 10.1093/geroni/igad104.3171

**Published:** 2023-12-21

**Authors:** Peace Amanambu, JohnBosco Chukwuorji, Eric Allard

**Affiliations:** University of Nigeria-Nsukka, Nsukka, Enugu, Nigeria; Michigan State University, East Lansing, Michigan, United States; Cleveland State University, Cleveland, Ohio, United States

## Abstract

The mediating role of mindfulness on the relationship between age and affect (both positive and negative) has been established in Western samples. However, these associations have not been well considered within additional cultural contexts. The present study examined whether mindfulness mediates the relationship between increased age and both positive (PA) and negative (NA) affect. We also took an exploratory approach to assess the strength and direction of the age-affect relationship as a function of resilience. Participants were 242 (95 women, age range = 18-75 years, Mage = 41.79; SDage = 13.58) adults, affiliated with the University of Nigeria-Nsukka, who completed the Positive and Negative Affect Scale, Brief Resilience Scale, and the Mindful Attention Awareness Scale through Qualtrics. Separate moderated-mediation analyses were conducted for PA and NA. For the PA model, the only significant association was between age and mindfulness, with higher age predicting increased self-reported mindfulness. For the NA model, higher self-reported mindfulness predicted lower levels of NA. When NA was simultaneously regressed on age and mindfulness, the direct effect between age and NA was non-significant, but the indirect effect of age on NA through mindfulness was significant, suggesting that the association between age and negative affect was mediated by mindfulness. Resilience did not provide a significant moderating influence in the PA and NA models. The present findings suggest that older adults may experience diminished levels of negative affect due to an increased capacity for mindfulness. Hence, mindfulness-based interventions may be helpful in improving the well-being of older populations.

